# Tetra­kis(μ-naphthalene-1-acetato-1:2κ^2^ *O*:*O*′)bis­(naphthalene-1-acetato)-1κ^2^ *O*,*O*′;2κ^2^ *O*,*O*′-bis­(1,10-phenanthroline)-1κ^2^ *N*,*N*′;2κ^2^ *N*,*N*′-europium(III)samarium(III)

**DOI:** 10.1107/S1600536808032960

**Published:** 2008-10-18

**Authors:** Hai-Tao Xia, Yu-Fen Liu, Liang Chen, Da-Qi Wang

**Affiliations:** a Department of Chemical Engineering, Huaihai Institute of Technology, Lianyungang Jiangsu 222005, People’s Republic of China; b College of Chemistry and Chemical Engineering, Liaocheng University, Shandong 252059, People’s Republic of China

## Abstract

In the title compound, [EuSm(C_12_H_9_O_2_)_6_(C_12_H_8_N_2_)_2_], the metal site is statistically occupied (50:50) by Eu and Sm atoms, forming a centrosymmetric complex. The metal site is nine-coordinate, in a distorted monocapped square-anti­prismatic coordination geometry. Mol­ecules are linked into three chains by C—H⋯π interactions and C—H⋯O hydrogen bonds. The combination of these chains generates a three-dimensional framework structure. One of the bridging naphthalene-1-ace­tate ligands was found to be disordered over two sites; the site occupancies for the naphthylmethyl group refined to 0.628 (14) and 0.372 (14).

## Related literature

For related structures, see: Liu *et al.* (2007 ▶); Xia *et al.* (2007 ▶).
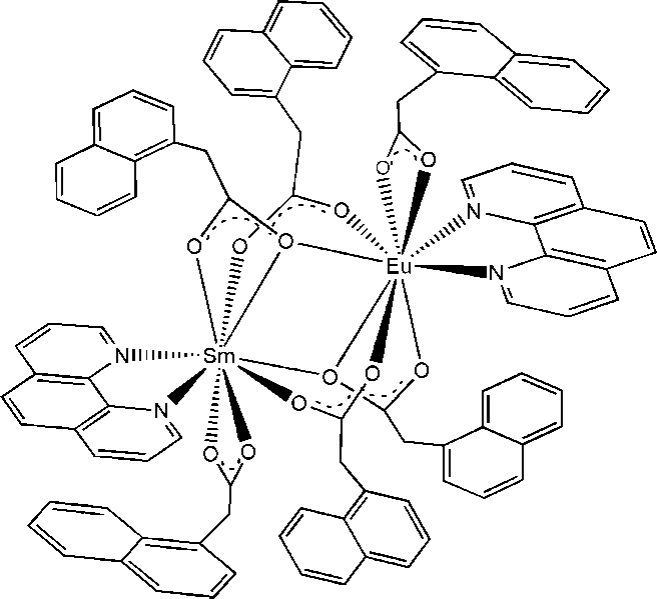


## Experimental

### 

#### Crystal data

[EuSm(C_12_H_9_O_2_)_6_(C_12_H_8_N_2_)_2_]
*M* *_r_* = 1773.87
Triclinic, 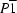

*a* = 11.9803 (18) Å
*b* = 12.4116 (19) Å
*c* = 15.041 (3) Å
α = 76.333 (3)°
β = 74.517 (3)°
γ = 66.768 (2)°
*V* = 1958.8 (5) Å^3^
*Z* = 1
Mo *K*α radiation
μ = 1.60 mm^−1^
*T* = 298 (2) K
0.28 × 0.19 × 0.15 mm

#### Data collection

Bruker SMART 1000 CCD area-detector diffractometer
Absorption correction: multi-scan (*SADABS*; Sheldrick, 1996 ▶) *T*_min_ = 0.662, *T*_max_ = 0.795
10253 measured reflections
6796 independent reflections
4983 reflections with *I* > 2σ(*I*)
*R*_int_ = 0.032

#### Refinement

*R*[*F* ^2^ > 2σ(*F* ^2^)] = 0.052
*wR*(*F* ^2^) = 0.128
*S* = 1.08
6796 reflections
608 parameters
H-atom parameters constrained
Δρ_max_ = 1.83 e Å^−3^
Δρ_min_ = −0.88 e Å^−3^

### 

Data collection: *SMART* (Siemens, 1996 ▶); cell refinement: *SAINT* (Siemens, 1996 ▶); data reduction: *SAINT*; program(s) used to solve structure: *SHELXS97* (Sheldrick, 2008 ▶); program(s) used to refine structure: *SHELXL97* (Sheldrick, 2008 ▶); molecular graphics: *SHELXTL* (Sheldrick, 2008 ▶); software used to prepare material for publication: *SHELXTL*.

## Supplementary Material

Crystal structure: contains datablocks I, global. DOI: 10.1107/S1600536808032960/at2644sup1.cif

Structure factors: contains datablocks I. DOI: 10.1107/S1600536808032960/at2644Isup2.hkl

Additional supplementary materials:  crystallographic information; 3D view; checkCIF report

## Figures and Tables

**Table 1:** Selected bond lengths (Å) *M* denotes Eu or Sm.

*M*—O3^i^	2.362 (4)
*M*—O1	2.385 (5)
*M*—O2^i^	2.396 (5)
*M*—O6	2.447 (5)
*M*—O5	2.479 (6)
*M*—O4	2.509 (5)
*M*—O3	2.570 (5)
*M*—N1	2.612 (6)
*M*—N2	2.627 (6)

Symmetry code: (i) 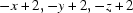.

**Table 2:** Hydrogen-bond geometry (Å, °)

*D*—H⋯*A*	*D*—H	H⋯*A*	*D*⋯*A*	*D*—H⋯*A*
C37—H37⋯O2^i^	0.93	2.45	3.097 (9)	127
C46—H46⋯O1	0.93	2.38	3.047 (10)	129
C11—H11⋯O5^ii^	0.93	2.48	3.38 (2)	163
C47—H47⋯O4^iii^	0.93	2.50	3.352 (10)	153
C35—H35⋯O6^iv^	0.93	2.67	3.327 (12)	128
C16—H16⋯*Cg*1^v^	0.93	2.87	3.66 (3)	144
C39—H39⋯*Cg*2^iii^	0.93	2.83	3.673 (18)	151

Symmetry codes: (i) 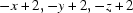; (ii) 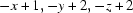; (iii) 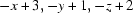; (iv) 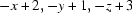; (v) 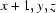. *Cg*1 and *Cg*2 are the centroids of the C7–C12 and C19–C24 rings, respectively.

**Fig. 1.**
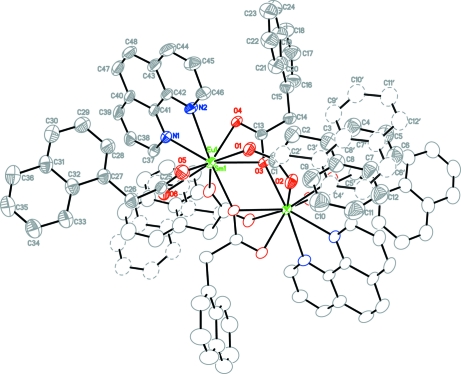
The molecular structure of (I), showing the atom-labelling scheme. Displacement ellipsoids are at the 30% probability level. Unlabelled atoms bonded to Sm or Eu are related to labelled atoms by (2 - x, 2 - y, 2 - z). For clarity, H atoms have been omitted.

**Fig. 2.**
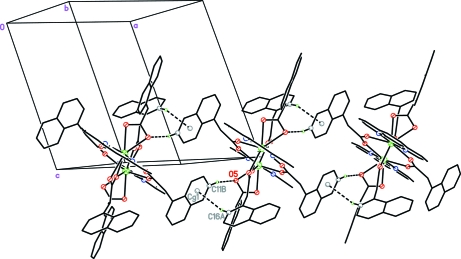
A larger portion of the crystal structure of (I), showing the formation of a hydrogen-bonded chain built from C—H···π and C—H···O. For clarity, H atoms not involved in the hydrogen bonding have been omitted. Dashed lines indicate hydrogen bonds [symmetry codes: (A) 2 - x, 2 - y, 2 - z; (B) 1 - x, 2 - y, 2 - z].

**Fig. 3.**
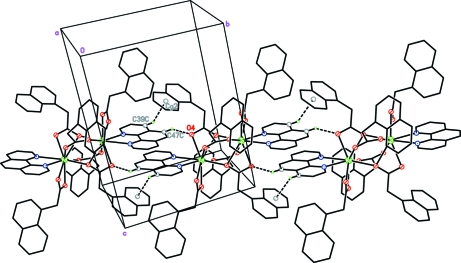
A larger portion of the crystal structure of (I), showing the formation of a hydrogen-bonded chain built from C—H···π and C—H···O. For clarity, H atoms not involved in the hydrogen bonding have been omitted. Dashed lines indicate hydrogen bonds [symmetry codes: (C) 3 - x, 1 - y, 2 - z].

**Fig. 4.**
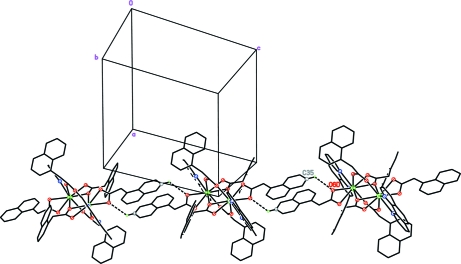
A larger portion of the crystal structure of (I), showing the formation of a hydrogen-bonded chain built from C—H···O. For clarity, H atoms not involved in the hydrogen bonding have been omitted. Dashed lines indicate hydrogen bonds [symmetry codes: (D) 2 - x, 1 - y, 3 - z].

## supplementary crystallographic information

### Comment

As a part of our investigation of the rare earth complexes with 1-naphthylacetic acid (NAA) and 1,10-phenanthroline (phen), we have recently reported the crystal structures of two complexes [Eu(NAA)_3_(phen)]_2_.2DMF(II) (Liu *et al.*, 2007 A T2403) and [Pr(NAA)_3_(phen)]_2 _.DMF (Xia *et al.*, 2007 A T2404). We report here the crystal structure of a new rare earth complexes with NAA and phen, (I).

In the title complex, the coordination environment of Sm and Eu atoms and coordination modes of the NNA ligands coordinated to the Sm^III^ and Eu^III^ ions are in agreement with the complex(II) (Fig.1). The average bond lengths of between the samarium and europium center and carboxylic oxygen atoms are 2.450 (5) Å, shorter than that (2.4725 (5) Å) of complex (II). The dihedral angles of the least-square-plane Sm_2_O_2 _or Eu_2_O_2 _and naphthyl rings are 31.05 (84)°(C3—C12 ring), 53.76 (33)°(C15—C24 ring) and 7.96 (53)°(C27—C36 ring).

The molecules of (I) are linked into three chains sheets by means of C—H···πand C—H···O hydrogen bond (Fig. 2, Fig. 3, Fig. 4 and Table 2). The action of three chains are to link complex into the three-dimensional framework structure. *Cg*1 and *Cg*2 are the centroids of the C7—C12 and C19—C24 ring, respectively.

The naphthalene-1-acetate ligand bridged to Sm and Eu were found to be disordered over two sites, the coordinates of these two sites were refined with the occupancies tied to sum to unity, the site occupancies for C2–C12 with attached H atoms and C2'–C12' with attached H atoms refined to 0.628 (14) and 0.372 (14), respectively.

### Experimental

To a stirred solution of 1-naphthylacetic acid (0.5586 g, 3 mmol) and 1,10-phenanthroline monohydrate (0.198 g, 1 mmol) in 30 ml methanol, and a solution of Sm(NO_3_)_3_.6H_2_O (0.182 g, 0.5 mmol) and Eu(NO_3_)_3_.6H_2_O (0.223 g, 0.5 mmol)in water (10 ml) was added. The mixed solution was heated to 333 K and stirred for 3 h, and then cooled to room temperature. The precipitate was washed with water and then dissolved in DMF. A colourless crystal suitable for X-ray diffraction was obtained by evaporation of DMF solution.

### Refinement

The space group was uniquely assigned from the systematic absences. All H atoms were located in difference Fourier maps. H atoms bonded to C atoms were treated as riding atoms, with C—H distances of 0.93 Å (aryl) and 0.97Å (methylene), and with *U*_iso_(H) = 1.2*U*_eq_(C) (aryl, methylene). The Sm and Eu atoms were found to be disordered over two positions, the occupancies of the two positions for Sm and Eu refined to 0.5 and 0.5. The NNA ligand bridged to Sm and Eu were found to be disordered over two sites, the coordinates of these two sites were refined with the occupancies tied to sum to unity, the site occupancies for C2—C12 with attached H atoms and C2'-C12' with attached H atoms refined to 0.628 (14) and 0.372 (14), respectively.

### Crystal data

**Table:**

[EuSm(C_12_H_9_O_2_)_6_(C_12_H_8_N_2_)_2_]	*Z* = 1
*M**_r_* = 1773.87	*F*(000) = 895
Triclinic, *P*1	*D*_x_ = 1.504 Mg m^−^^3^
Hall symbol: -P 1	Mo *K*α radiation, λ = 0.71073 Å
*a* = 11.9803 (18) Å	Cell parameters from 3440 reflections
*b* = 12.4116 (19) Å	θ = 2.6–25.1°
*c* = 15.041 (3) Å	µ = 1.60 mm^−^^1^
α = 76.333 (3)°	*T* = 298 K
β = 74.517 (3)°	Block, colourless
γ = 66.768 (2)°	0.28 × 0.19 × 0.15 mm
*V* = 1958.8 (5) Å^3^	

### Data collection

**Table:**

Bruker SMART 1000 CCD area-detector diffractometer	6796 independent reflections
Radiation source: fine-focus sealed tube	4983 reflections with *I* > 2σ(*I*)
graphite	*R*_int_ = 0.032
φ and ω scans	θ_max_ = 25.0°, θ_min_ = 1.4°
Absorption correction: multi-scan (SADABS; Sheldrick, 1996)	*h* = −14→14
*T*_min_ = 0.662, *T*_max_ = 0.795	*k* = −14→9
10253 measured reflections	*l* = −17→16

### Refinement

**Table:**

Refinement on *F*^2^	Primary atom site location: structure-invariant direct methods
Least-squares matrix: full	Secondary atom site location: difference Fourier map
*R*[*F*^2^ > 2σ(*F*^2^)] = 0.052	Hydrogen site location: inferred from neighbouring sites
*wR*(*F*^2^) = 0.128	H-atom parameters constrained
*S* = 1.08	*w* = 1/[σ^2^(*F*_o_^2^) + (0.0343*P*)^2^ + 6.8621*P*] where *P* = (*F*_o_^2^ + 2*F*_c_^2^)/3
6796 reflections	(Δ/σ)_max_ = 0.001
608 parameters	Δρ_max_ = 1.83 e Å^−^^3^
0 restraints	Δρ_min_ = −0.88 e Å^−^^3^

### Special details

**Table:**

Geometry. All e.s.d.'s (except the e.s.d. in the dihedral angle between two l.s. planes) are estimated using the full covariance matrix. The cell e.s.d.'s are taken into account individually in the estimation of e.s.d.'s in distances, angles and torsion angles; correlations between e.s.d.'s in cell parameters are only used when they are defined by crystal symmetry. An approximate (isotropic) treatment of cell e.s.d.'s is used for estimating e.s.d.'s involving l.s. planes.
Refinement. Refinement of *F*^2^ against ALL reflections. The weighted *R*-factor *wR* and goodness of fit *S* are based on *F*^2^, conventional *R*-factors *R* are based on *F*, with *F* set to zero for negative *F*^2^. The threshold expression of *F*^2^ > σ(*F*^2^) is used only for calculating *R*-factors(gt) *etc*. and is not relevant to the choice of reflections for refinement. *R*-factors based on *F*^2^ are statistically about twice as large as those based on *F*, and *R*- factors based on ALL data will be even larger.

### Fractional atomic coordinates and isotropic or equivalent isotropic displacement parameters (Å^2^)

**Table:**

	*x*	*y*	*z*	*U*_iso_*/*U*_eq_	Occ. (<1)
Sm1	1.07826 (4)	0.83074 (3)	1.04773 (3)	0.04137 (14)	0.50
Eu1	1.07826 (4)	0.83074 (3)	1.04773 (3)	0.04137 (14)	0.50
N1	1.2755 (5)	0.6709 (5)	1.1043 (4)	0.0494 (15)	
N2	1.1592 (6)	0.6211 (5)	0.9953 (4)	0.0523 (16)	
O1	0.9538 (5)	0.8600 (4)	0.9379 (4)	0.0596 (14)	
O2	0.8419 (5)	1.0516 (4)	0.8993 (4)	0.0586 (14)	
O3	1.0983 (4)	1.0007 (4)	0.9160 (3)	0.0479 (12)	
O4	1.2420 (5)	0.8254 (4)	0.9026 (3)	0.0546 (14)	
O5	0.9310 (5)	0.7296 (5)	1.1352 (4)	0.0670 (16)	
O6	1.0279 (5)	0.7739 (5)	1.2167 (4)	0.0621 (15)	
C1	0.8748 (9)	0.9432 (8)	0.8980 (6)	0.069 (3)	
C2	0.843 (2)	0.8996 (15)	0.8199 (15)	0.082 (6)	0.628 (14)
H2A	0.9206	0.8573	0.7816	0.098*	0.628 (14)
H2B	0.8023	0.8432	0.8505	0.098*	0.628 (14)
C3	0.762 (4)	0.995 (3)	0.755 (3)	0.084 (10)	0.628 (14)
C4	0.818 (3)	1.042 (2)	0.6701 (19)	0.098 (7)	0.628 (14)
H4	0.9035	1.0132	0.6515	0.117*	0.628 (14)
C5	0.745 (3)	1.132 (3)	0.613 (3)	0.102 (8)	0.628 (14)
H5	0.7826	1.1638	0.5555	0.122*	0.628 (14)
C6	0.617 (4)	1.175 (4)	0.640 (3)	0.100 (11)	0.628 (14)
H6	0.5688	1.2357	0.6020	0.120*	0.628 (14)
C7	0.562 (7)	1.128 (4)	0.726 (4)	0.093 (14)	0.628 (14)
C8	0.634 (4)	1.038 (3)	0.783 (2)	0.081 (8)	0.628 (14)
C9	0.578 (3)	0.991 (3)	0.869 (2)	0.096 (8)	0.628 (14)
H9	0.6268	0.9302	0.9071	0.115*	0.628 (14)
C10	0.450 (2)	1.0336 (19)	0.8965 (16)	0.112 (7)	0.628 (14)
H10	0.4130	1.0020	0.9536	0.134*	0.628 (14)
C11	0.378 (2)	1.1238 (19)	0.8390 (18)	0.109 (7)	0.628 (14)
H11	0.2921	1.1526	0.8576	0.131*	0.628 (14)
C12	0.433 (3)	1.171 (2)	0.754 (3)	0.102 (9)	0.628 (14)
H12	0.3850	1.2315	0.7152	0.123*	0.628 (14)
C2'	0.766 (3)	0.906 (3)	0.882 (2)	0.081 (9)	0.372 (14)
H2'1	0.8018	0.8301	0.8602	0.097*	0.372 (14)
H2'2	0.7091	0.8990	0.9412	0.097*	0.372 (14)
C3'	0.694 (6)	1.001 (4)	0.810 (4)	0.088 (15)	0.372 (14)
C4'	0.568 (6)	1.060 (4)	0.840 (5)	0.093 (14)	0.372 (14)
H4'	0.5309	1.0457	0.9018	0.112*	0.372 (14)
C5'	0.497 (8)	1.139 (6)	0.776 (6)	0.101 (19)	0.372 (14)
H5'	0.4130	1.1782	0.7956	0.121*	0.372 (14)
C6'	0.553 (12)	1.160 (8)	0.683 (6)	0.10 (3)	0.372 (14)
H6'	0.5057	1.2132	0.6405	0.118*	0.372 (14)
C7'	0.679 (7)	1.102 (5)	0.654 (4)	0.099 (14)	0.372 (14)
C8'	0.750 (7)	1.022 (6)	0.718 (6)	0.102 (8)	0.372 (14)
C9'	0.876 (4)	0.964 (4)	0.688 (3)	0.092 (11)	0.372 (14)
H9'	0.9229	0.9111	0.7308	0.110*	0.372 (14)
C10'	0.931 (3)	0.985 (3)	0.595 (3)	0.104 (11)	0.372 (14)
H10'	1.0154	0.9461	0.5757	0.125*	0.372 (14)
C11'	0.860 (4)	1.064 (3)	0.532 (3)	0.104 (11)	0.372 (14)
H11'	0.8976	1.0785	0.4695	0.125*	0.372 (14)
C12'	0.734 (4)	1.123 (3)	0.561 (3)	0.095 (13)	0.372 (14)
H12'	0.6872	1.1759	0.5183	0.114*	0.372 (14)
C13	1.1879 (7)	0.9282 (7)	0.8676 (5)	0.0476 (18)	
C14	1.2204 (8)	0.9723 (7)	0.7647 (6)	0.068 (2)	
H14A	1.1490	0.9908	0.7375	0.082*	
H14B	1.2340	1.0459	0.7590	0.082*	
C15	1.3301 (9)	0.8920 (8)	0.7076 (6)	0.070 (2)	
C16	1.4319 (10)	0.9214 (10)	0.6720 (8)	0.094 (3)	
H16	1.4323	0.9900	0.6867	0.112*	
C17	1.5371 (12)	0.8548 (12)	0.6142 (9)	0.113 (4)	
H17	1.6058	0.8777	0.5913	0.136*	
C18	1.5348 (12)	0.7552 (12)	0.5927 (8)	0.110 (4)	
H18	1.6041	0.7089	0.5551	0.132*	
C19	1.4310 (11)	0.7202 (10)	0.6254 (7)	0.083 (3)	
C20	1.3258 (9)	0.7906 (9)	0.6834 (6)	0.072 (3)	
C21	1.2229 (10)	0.7551 (10)	0.7136 (7)	0.082 (3)	
H21	1.1526	0.7992	0.7518	0.099*	
C22	1.2249 (12)	0.6548 (11)	0.6874 (8)	0.099 (3)	
H22	1.1559	0.6321	0.7072	0.119*	
C23	1.3300 (14)	0.5884 (12)	0.6315 (9)	0.108 (4)	
H23	1.3308	0.5201	0.6156	0.130*	
C24	1.4283 (14)	0.6184 (12)	0.6003 (8)	0.108 (4)	
H24	1.4965	0.5728	0.5615	0.129*	
C25	0.9553 (8)	0.7250 (7)	1.2119 (6)	0.057 (2)	
C26	0.8989 (8)	0.6632 (8)	1.3013 (6)	0.074 (3)	
H26A	0.8168	0.6703	1.2969	0.089*	
H26B	0.8901	0.7020	1.3529	0.089*	
C27	0.9768 (8)	0.5329 (8)	1.3208 (6)	0.065 (2)	
C28	1.0766 (9)	0.4782 (8)	1.2587 (7)	0.073 (3)	
H28	1.0975	0.5213	1.2014	0.087*	
C29	1.1493 (10)	0.3589 (9)	1.2779 (8)	0.087 (3)	
H29	1.2186	0.3234	1.2347	0.104*	
C30	1.1168 (10)	0.2952 (9)	1.3615 (8)	0.084 (3)	
H30	1.1644	0.2154	1.3742	0.101*	
C31	1.0161 (10)	0.3454 (9)	1.4274 (7)	0.073 (3)	
C32	0.9429 (9)	0.4671 (8)	1.4084 (6)	0.066 (2)	
C33	0.8409 (9)	0.5175 (9)	1.4775 (7)	0.078 (3)	
H33	0.7922	0.5972	1.4663	0.093*	
C34	0.8125 (10)	0.4506 (11)	1.5611 (7)	0.089 (3)	
H34	0.7444	0.4846	1.6057	0.107*	
C35	0.8843 (12)	0.3342 (11)	1.5785 (8)	0.097 (3)	
H35	0.8640	0.2904	1.6356	0.116*	
C36	0.9831 (11)	0.2804 (10)	1.5165 (8)	0.093 (3)	
H36	1.0302	0.2009	1.5312	0.111*	
C37	1.3295 (7)	0.6925 (7)	1.1601 (6)	0.059 (2)	
H37	1.2984	0.7680	1.1768	0.071*	
C38	1.4318 (8)	0.6071 (8)	1.1957 (6)	0.071 (3)	
H38	1.4674	0.6263	1.2348	0.085*	
C39	1.4777 (8)	0.4974 (8)	1.1727 (6)	0.074 (3)	
H39	1.5464	0.4407	1.1951	0.088*	
C40	1.4237 (7)	0.4685 (7)	1.1161 (6)	0.059 (2)	
C41	1.3198 (7)	0.5590 (6)	1.0821 (5)	0.052 (2)	
C42	1.2593 (7)	0.5333 (6)	1.0247 (5)	0.052 (2)	
C43	1.3026 (8)	0.4186 (7)	1.0016 (6)	0.063 (2)	
C44	1.2385 (9)	0.3975 (8)	0.9463 (7)	0.079 (3)	
H44	1.2648	0.3226	0.9293	0.094*	
C45	1.1390 (9)	0.4843 (8)	0.9172 (7)	0.073 (3)	
H45	1.0959	0.4704	0.8806	0.087*	
C46	1.1024 (8)	0.5956 (7)	0.9435 (6)	0.065 (2)	
H46	1.0338	0.6554	0.9233	0.078*	
C47	1.4646 (9)	0.3538 (8)	1.0900 (7)	0.075 (3)	
H47	1.5320	0.2938	1.1118	0.090*	
C48	1.4090 (9)	0.3303 (8)	1.0353 (7)	0.075 (3)	
H48	1.4397	0.2549	1.0185	0.090*	

### Atomic displacement parameters (Å^2^)

**Table:**

	*U*^11^	*U*^22^	*U*^33^	*U*^12^	*U*^13^	*U*^23^
Sm1	0.0466 (2)	0.0276 (2)	0.0416 (2)	−0.00271 (15)	−0.01227 (16)	−0.00413 (14)
Eu1	0.0466 (2)	0.0276 (2)	0.0416 (2)	−0.00271 (15)	−0.01227 (16)	−0.00413 (14)
N1	0.047 (4)	0.041 (4)	0.049 (4)	−0.003 (3)	−0.013 (3)	−0.004 (3)
N2	0.059 (4)	0.033 (3)	0.059 (4)	−0.010 (3)	−0.007 (3)	−0.012 (3)
O1	0.073 (4)	0.040 (3)	0.067 (4)	−0.007 (3)	−0.036 (3)	−0.006 (3)
O2	0.075 (4)	0.037 (3)	0.077 (4)	−0.016 (3)	−0.043 (3)	−0.005 (3)
O3	0.049 (3)	0.033 (3)	0.048 (3)	0.000 (2)	−0.006 (2)	−0.008 (2)
O4	0.057 (3)	0.033 (3)	0.051 (3)	0.002 (2)	−0.005 (3)	−0.004 (2)
O5	0.081 (4)	0.059 (4)	0.063 (4)	−0.030 (3)	−0.009 (3)	−0.009 (3)
O6	0.062 (4)	0.060 (4)	0.049 (3)	−0.012 (3)	−0.012 (3)	0.002 (3)
C1	0.088 (7)	0.054 (6)	0.083 (7)	−0.024 (5)	−0.049 (6)	−0.008 (5)
C2	0.097 (14)	0.066 (10)	0.099 (15)	−0.019 (10)	−0.054 (12)	−0.019 (10)
C3	0.10 (2)	0.069 (15)	0.10 (3)	−0.028 (17)	−0.06 (2)	−0.017 (17)
C4	0.11 (2)	0.084 (18)	0.11 (2)	−0.025 (17)	−0.047 (18)	−0.013 (15)
C5	0.12 (2)	0.085 (16)	0.11 (2)	−0.028 (17)	−0.047 (19)	−0.008 (15)
C6	0.11 (3)	0.08 (2)	0.11 (3)	−0.02 (2)	−0.06 (2)	−0.007 (19)
C7	0.11 (4)	0.08 (2)	0.11 (5)	−0.03 (3)	−0.06 (3)	−0.01 (3)
C8	0.10 (2)	0.068 (15)	0.10 (2)	−0.025 (14)	−0.06 (2)	−0.019 (15)
C9	0.11 (2)	0.076 (17)	0.11 (2)	−0.019 (16)	−0.046 (16)	−0.017 (14)
C10	0.122 (19)	0.098 (16)	0.117 (17)	−0.025 (15)	−0.040 (15)	−0.026 (13)
C11	0.119 (19)	0.090 (15)	0.12 (2)	−0.017 (13)	−0.045 (16)	−0.035 (14)
C12	0.12 (2)	0.084 (16)	0.11 (2)	−0.018 (16)	−0.06 (2)	−0.020 (14)
C2'	0.10 (2)	0.066 (18)	0.09 (2)	−0.027 (18)	−0.051 (19)	−0.013 (17)
C3'	0.11 (4)	0.07 (2)	0.10 (4)	−0.03 (3)	−0.05 (4)	−0.02 (3)
C4'	0.11 (4)	0.08 (3)	0.11 (4)	−0.02 (3)	−0.05 (3)	−0.01 (3)
C5'	0.11 (6)	0.08 (3)	0.11 (6)	−0.02 (4)	−0.06 (4)	−0.02 (3)
C6'	0.11 (7)	0.08 (5)	0.11 (9)	−0.03 (5)	−0.05 (6)	−0.01 (5)
C7'	0.11 (4)	0.08 (3)	0.11 (4)	−0.02 (3)	−0.05 (4)	−0.01 (3)
C8'	0.12 (2)	0.085 (16)	0.11 (2)	−0.028 (17)	−0.047 (19)	−0.008 (15)
C9'	0.11 (3)	0.07 (3)	0.10 (3)	−0.03 (2)	−0.05 (3)	−0.01 (2)
C10'	0.12 (3)	0.08 (2)	0.11 (3)	−0.03 (2)	−0.05 (2)	−0.02 (2)
C11'	0.12 (3)	0.09 (2)	0.11 (3)	−0.03 (2)	−0.05 (2)	−0.01 (2)
C12'	0.12 (3)	0.08 (2)	0.10 (3)	−0.03 (2)	−0.06 (3)	−0.02 (2)
C13	0.050 (5)	0.038 (4)	0.052 (5)	−0.012 (4)	−0.006 (4)	−0.013 (4)
C14	0.073 (6)	0.051 (5)	0.056 (5)	−0.009 (4)	0.006 (4)	−0.009 (4)
C15	0.072 (6)	0.064 (6)	0.063 (6)	−0.018 (5)	0.001 (5)	−0.014 (5)
C16	0.088 (8)	0.084 (8)	0.096 (8)	−0.029 (7)	0.009 (7)	−0.026 (6)
C17	0.096 (9)	0.111 (10)	0.110 (10)	−0.026 (8)	0.012 (7)	−0.029 (8)
C18	0.093 (9)	0.101 (10)	0.100 (9)	−0.005 (8)	0.006 (7)	−0.028 (8)
C19	0.092 (8)	0.080 (8)	0.072 (7)	−0.017 (6)	−0.015 (6)	−0.026 (6)
C20	0.079 (7)	0.069 (6)	0.063 (6)	−0.015 (5)	−0.015 (5)	−0.016 (5)
C21	0.086 (8)	0.082 (8)	0.075 (7)	−0.022 (6)	−0.017 (6)	−0.017 (6)
C22	0.105 (9)	0.099 (9)	0.096 (9)	−0.032 (8)	−0.024 (7)	−0.021 (7)
C23	0.124 (11)	0.106 (10)	0.095 (10)	−0.024 (9)	−0.030 (9)	−0.032 (8)
C24	0.113 (11)	0.103 (10)	0.081 (8)	−0.006 (8)	−0.015 (8)	−0.028 (7)
C25	0.053 (5)	0.040 (5)	0.059 (6)	−0.009 (4)	−0.001 (4)	0.002 (4)
C26	0.071 (6)	0.063 (6)	0.065 (6)	−0.019 (5)	0.004 (5)	0.003 (5)
C27	0.069 (6)	0.059 (6)	0.064 (6)	−0.028 (5)	−0.009 (5)	−0.001 (4)
C28	0.074 (6)	0.064 (6)	0.068 (6)	−0.024 (5)	0.001 (5)	−0.004 (5)
C29	0.085 (7)	0.070 (7)	0.089 (8)	−0.018 (6)	−0.005 (6)	−0.011 (6)
C30	0.091 (8)	0.070 (7)	0.087 (8)	−0.026 (6)	−0.027 (6)	0.001 (6)
C31	0.087 (7)	0.070 (7)	0.071 (6)	−0.042 (6)	−0.022 (6)	0.007 (5)
C32	0.074 (6)	0.069 (6)	0.064 (6)	−0.039 (5)	−0.015 (5)	−0.001 (5)
C33	0.083 (7)	0.082 (7)	0.068 (6)	−0.037 (6)	−0.013 (5)	0.001 (5)
C34	0.091 (8)	0.098 (9)	0.076 (7)	−0.046 (7)	−0.008 (6)	0.000 (6)
C35	0.112 (10)	0.098 (9)	0.075 (8)	−0.046 (8)	−0.021 (7)	0.015 (7)
C36	0.106 (9)	0.080 (8)	0.088 (8)	−0.039 (7)	−0.024 (7)	0.010 (6)
C37	0.053 (5)	0.054 (5)	0.059 (5)	−0.004 (4)	−0.020 (4)	−0.006 (4)
C38	0.059 (6)	0.072 (7)	0.065 (6)	−0.005 (5)	−0.025 (5)	0.001 (5)
C39	0.056 (6)	0.059 (6)	0.072 (6)	0.002 (5)	−0.014 (5)	0.014 (5)
C40	0.048 (5)	0.040 (5)	0.062 (5)	−0.001 (4)	−0.004 (4)	0.007 (4)
C41	0.047 (5)	0.038 (4)	0.051 (5)	−0.005 (4)	−0.001 (4)	0.004 (3)
C42	0.051 (5)	0.033 (4)	0.056 (5)	−0.008 (4)	0.002 (4)	−0.004 (3)
C43	0.063 (6)	0.037 (5)	0.073 (6)	−0.013 (4)	0.002 (5)	−0.006 (4)
C44	0.083 (7)	0.043 (5)	0.089 (7)	−0.008 (5)	0.005 (6)	−0.019 (5)
C45	0.083 (7)	0.055 (6)	0.085 (7)	−0.026 (5)	−0.010 (5)	−0.025 (5)
C46	0.071 (6)	0.048 (5)	0.076 (6)	−0.018 (4)	−0.014 (5)	−0.017 (4)
C47	0.064 (6)	0.045 (6)	0.081 (7)	−0.001 (5)	0.000 (5)	0.007 (5)
C48	0.072 (6)	0.038 (5)	0.083 (7)	−0.003 (5)	0.009 (5)	−0.009 (5)

### Geometric parameters (Å, °)

**Table:**

Sm1—O3^i^	2.362 (4)	C11'—H11'	0.9300
Sm1—O1	2.385 (5)	C12'—H12'	0.9300
Sm1—O2^i^	2.396 (5)	C13—C14	1.514 (10)
Sm1—O6	2.447 (5)	C14—C15	1.492 (11)
Sm1—O5	2.479 (6)	C14—H14A	0.9700
Sm1—O4	2.509 (5)	C14—H14B	0.9700
Sm1—O3	2.570 (5)	C15—C16	1.348 (13)
Sm1—N1	2.612 (6)	C15—C20	1.414 (13)
Sm1—N2	2.627 (6)	C16—C17	1.400 (14)
Sm1—Eu1^i^	3.9500 (9)	C16—H16	0.9300
Sm1—Sm1^i^	3.9500 (9)	C17—C18	1.361 (16)
N1—C37	1.314 (9)	C17—H17	0.9300
N1—C41	1.366 (9)	C18—C19	1.406 (15)
N2—C46	1.320 (10)	C18—H18	0.9300
N2—C42	1.358 (9)	C19—C24	1.417 (16)
O1—C1	1.250 (9)	C19—C20	1.419 (13)
O2—C1	1.247 (9)	C20—C21	1.399 (13)
O2—Sm1^i^	2.396 (5)	C21—C22	1.381 (14)
O2—Eu1^i^	2.396 (5)	C21—H21	0.9300
O3—C13	1.274 (8)	C22—C23	1.384 (16)
O3—Eu1^i^	2.362 (4)	C22—H22	0.9300
O3—Sm1^i^	2.362 (4)	C23—C24	1.310 (16)
O4—C13	1.240 (8)	C23—H23	0.9300
O5—C25	1.248 (10)	C24—H24	0.9300
O6—C25	1.264 (10)	C25—C26	1.513 (11)
C1—C2	1.586 (17)	C26—C27	1.518 (12)
C1—C2'	1.63 (3)	C26—H26A	0.9700
C2—C3	1.53 (3)	C26—H26B	0.9700
C2—H2A	0.9700	C27—C28	1.351 (12)
C2—H2B	0.9700	C27—C32	1.426 (11)
C3—C4	1.39 (5)	C28—C29	1.396 (12)
C3—C8	1.39 (6)	C28—H28	0.9300
C4—C5	1.39 (4)	C29—C30	1.367 (13)
C4—H4	0.9300	C29—H29	0.9300
C5—C6	1.39 (5)	C30—C31	1.374 (13)
C5—H5	0.9300	C30—H30	0.9300
C6—C7	1.39 (8)	C31—C32	1.421 (13)
C6—H6	0.9300	C31—C36	1.435 (13)
C7—C8	1.39 (6)	C32—C33	1.411 (12)
C7—C12	1.39 (9)	C33—C34	1.374 (12)
C8—C9	1.39 (5)	C33—H33	0.9300
C9—C10	1.39 (4)	C34—C35	1.362 (14)
C9—H9	0.9300	C34—H34	0.9300
C10—C11	1.39 (3)	C35—C36	1.341 (15)
C10—H10	0.9300	C35—H35	0.9300
C11—C12	1.39 (4)	C36—H36	0.9300
C11—H11	0.9300	C37—C38	1.404 (10)
C12—H12	0.9300	C37—H37	0.9300
C2'—C3'	1.55 (6)	C38—C39	1.344 (12)
C2'—H2'1	0.9700	C38—H38	0.9300
C2'—H2'2	0.9700	C39—C40	1.377 (12)
C3'—C4'	1.39 (8)	C39—H39	0.9300
C3'—C8'	1.39 (11)	C40—C47	1.426 (12)
C4'—C5'	1.39 (9)	C40—C41	1.427 (10)
C4'—H4'	0.9300	C41—C42	1.421 (11)
C5'—C6'	1.39 (14)	C42—C43	1.404 (11)
C5'—H5'	0.9300	C43—C44	1.399 (13)
C6'—C7'	1.39 (15)	C43—C48	1.437 (12)
C6'—H6'	0.9300	C44—C45	1.348 (12)
C7'—C12'	1.39 (8)	C44—H44	0.9300
C7'—C8'	1.39 (8)	C45—C46	1.394 (11)
C8'—C9'	1.39 (9)	C45—H45	0.9300
C9'—C10'	1.39 (5)	C46—H46	0.9300
C9'—H9'	0.9300	C47—C48	1.328 (13)
C10'—C11'	1.39 (4)	C47—H47	0.9300
C10'—H10'	0.9300	C48—H48	0.9300
C11'—C12'	1.39 (5)		
			
O3^i^—Sm1—O1	75.35 (17)	C6'—C7'—C8'	120 (7)
O3^i^—Sm1—O2^i^	76.63 (18)	C12'—C7'—C8'	120 (7)
O1—Sm1—O2^i^	136.94 (17)	C3'—C8'—C9'	120 (6)
O3^i^—Sm1—O6	80.85 (17)	C3'—C8'—C7'	120 (7)
O1—Sm1—O6	126.6 (2)	C9'—C8'—C7'	120 (8)
O2^i^—Sm1—O6	79.5 (2)	C10'—C9'—C8'	120 (5)
O3^i^—Sm1—O5	81.14 (18)	C10'—C9'—H9'	120.0
O1—Sm1—O5	76.8 (2)	C8'—C9'—H9'	120.0
O2^i^—Sm1—O5	129.6 (2)	C9'—C10'—C11'	120 (4)
O6—Sm1—O5	52.4 (2)	C9'—C10'—H10'	120.0
O3^i^—Sm1—O4	124.70 (16)	C11'—C10'—H10'	120.0
O1—Sm1—O4	82.14 (18)	C10'—C11'—C12'	120 (4)
O2^i^—Sm1—O4	87.72 (19)	C10'—C11'—H11'	120.0
O6—Sm1—O4	147.99 (18)	C12'—C11'—H11'	120.0
O5—Sm1—O4	140.92 (18)	C7'—C12'—C11'	120 (5)
O3^i^—Sm1—O3	73.64 (18)	C7'—C12'—H12'	120.0
O1—Sm1—O3	69.34 (17)	C11'—C12'—H12'	120.0
O2^i^—Sm1—O3	71.77 (17)	O4—C13—O3	121.4 (7)
O6—Sm1—O3	145.08 (18)	O4—C13—C14	122.0 (7)
O5—Sm1—O3	141.76 (18)	O3—C13—C14	116.5 (7)
O4—Sm1—O3	51.14 (15)	C15—C14—C13	117.6 (7)
O3^i^—Sm1—N1	144.94 (18)	C15—C14—H14A	107.9
O1—Sm1—N1	138.67 (19)	C13—C14—H14A	107.9
O2^i^—Sm1—N1	78.07 (19)	C15—C14—H14B	107.9
O6—Sm1—N1	70.97 (18)	C13—C14—H14B	107.9
O5—Sm1—N1	97.0 (2)	H14A—C14—H14B	107.2
O4—Sm1—N1	77.75 (17)	C16—C15—C20	119.0 (9)
O3—Sm1—N1	120.13 (17)	C16—C15—C14	119.6 (9)
O3^i^—Sm1—N2	145.13 (19)	C20—C15—C14	121.2 (9)
O1—Sm1—N2	76.94 (19)	C15—C16—C17	123.8 (11)
O2^i^—Sm1—N2	138.0 (2)	C15—C16—H16	118.1
O6—Sm1—N2	99.40 (19)	C17—C16—H16	118.1
O5—Sm1—N2	72.25 (19)	C18—C17—C16	117.5 (12)
O4—Sm1—N2	71.10 (18)	C18—C17—H17	121.3
O3—Sm1—N2	115.18 (17)	C16—C17—H17	121.3
N1—Sm1—N2	62.5 (2)	C17—C18—C19	122.0 (12)
O3^i^—Sm1—Eu1^i^	38.63 (11)	C17—C18—H18	119.0
O1—Sm1—Eu1^i^	67.62 (12)	C19—C18—H18	119.0
O2^i^—Sm1—Eu1^i^	70.01 (12)	C18—C19—C24	121.5 (12)
O6—Sm1—Eu1^i^	116.00 (13)	C18—C19—C20	118.8 (11)
O5—Sm1—Eu1^i^	114.77 (14)	C24—C19—C20	119.6 (11)
O4—Sm1—Eu1^i^	86.11 (11)	C21—C20—C15	123.5 (9)
O3—Sm1—Eu1^i^	35.01 (10)	C21—C20—C19	117.7 (10)
N1—Sm1—Eu1^i^	144.69 (14)	C15—C20—C19	118.8 (10)
N2—Sm1—Eu1^i^	140.12 (14)	C22—C21—C20	120.5 (11)
O3^i^—Sm1—Sm1^i^	38.63 (11)	C22—C21—H21	119.7
O1—Sm1—Sm1^i^	67.62 (12)	C20—C21—H21	119.7
O2^i^—Sm1—Sm1^i^	70.01 (12)	C21—C22—C23	119.7 (12)
O6—Sm1—Sm1^i^	116.00 (13)	C21—C22—H22	120.1
O5—Sm1—Sm1^i^	114.77 (14)	C23—C22—H22	120.1
O4—Sm1—Sm1^i^	86.11 (11)	C24—C23—C22	122.3 (14)
O3—Sm1—Sm1^i^	35.01 (10)	C24—C23—H23	118.9
N1—Sm1—Sm1^i^	144.69 (14)	C22—C23—H23	118.9
N2—Sm1—Sm1^i^	140.12 (14)	C23—C24—C19	120.1 (13)
Eu1^i^—Sm1—Sm1^i^	0.000 (14)	C23—C24—H24	119.9
C37—N1—C41	118.0 (7)	C19—C24—H24	119.9
C37—N1—Sm1	121.5 (5)	O5—C25—O6	120.0 (7)
C41—N1—Sm1	120.4 (5)	O5—C25—C26	121.7 (9)
C46—N2—C42	117.8 (7)	O6—C25—C26	118.2 (9)
C46—N2—Sm1	122.0 (5)	C25—C26—C27	112.2 (7)
C42—N2—Sm1	120.2 (5)	C25—C26—H26A	109.2
C1—O1—Sm1	138.5 (5)	C27—C26—H26A	109.2
C1—O2—Sm1^i^	133.7 (5)	C25—C26—H26B	109.2
C1—O2—Eu1^i^	133.7 (5)	C27—C26—H26B	109.2
C13—O3—Eu1^i^	159.7 (5)	H26A—C26—H26B	107.9
C13—O3—Sm1^i^	159.7 (5)	C28—C27—C32	119.5 (9)
C13—O3—Sm1	90.9 (4)	C28—C27—C26	122.4 (8)
Eu1^i^—O3—Sm1	106.36 (18)	C32—C27—C26	118.2 (8)
Sm1^i^—O3—Sm1	106.36 (18)	C27—C28—C29	122.0 (9)
C13—O4—Sm1	94.6 (4)	C27—C28—H28	119.0
C25—O5—Sm1	93.1 (5)	C29—C28—H28	119.0
C25—O6—Sm1	94.2 (5)	C30—C29—C28	118.8 (10)
O2—C1—O1	128.1 (8)	C30—C29—H29	120.6
O2—C1—C2	119.5 (9)	C28—C29—H29	120.6
O1—C1—C2	110.6 (9)	C29—C30—C31	122.0 (10)
O2—C1—C2'	112.8 (13)	C29—C30—H30	119.0
O1—C1—C2'	113.6 (12)	C31—C30—H30	119.0
C3—C2—C1	116.9 (16)	C30—C31—C32	119.2 (9)
C3—C2—H2A	108.1	C30—C31—C36	122.6 (10)
C1—C2—H2A	108.1	C32—C31—C36	118.2 (10)
C3—C2—H2B	108.1	C33—C32—C31	118.4 (9)
C1—C2—H2B	108.1	C33—C32—C27	123.1 (9)
H2A—C2—H2B	107.3	C31—C32—C27	118.5 (9)
C4—C3—C8	120 (3)	C34—C33—C32	120.9 (10)
C4—C3—C2	119 (3)	C34—C33—H33	119.5
C8—C3—C2	121 (4)	C32—C33—H33	119.5
C3—C4—C5	120 (4)	C35—C34—C33	119.9 (11)
C3—C4—H4	120.0	C35—C34—H34	120.1
C5—C4—H4	120.0	C33—C34—H34	120.1
C4—C5—C6	120 (4)	C36—C35—C34	122.6 (11)
C4—C5—H5	120.0	C36—C35—H35	118.7
C6—C5—H5	120.0	C34—C35—H35	118.7
C5—C6—C7	120 (4)	C35—C36—C31	120.0 (11)
C5—C6—H6	120.0	C35—C36—H36	120.0
C7—C6—H6	120.0	C31—C36—H36	120.0
C8—C7—C12	120 (6)	N1—C37—C38	123.1 (8)
C8—C7—C6	120 (6)	N1—C37—H37	118.4
C12—C7—C6	120 (4)	C38—C37—H37	118.4
C3—C8—C7	120 (4)	C39—C38—C37	119.3 (9)
C3—C8—C9	120 (4)	C39—C38—H38	120.3
C7—C8—C9	120 (5)	C37—C38—H38	120.3
C10—C9—C8	120 (3)	C38—C39—C40	120.3 (8)
C10—C9—H9	120.0	C38—C39—H39	119.8
C8—C9—H9	120.0	C40—C39—H39	119.8
C11—C10—C9	120 (2)	C39—C40—C47	124.0 (8)
C11—C10—H10	120.0	C39—C40—C41	117.8 (8)
C9—C10—H10	120.0	C47—C40—C41	118.2 (9)
C10—C11—C12	120 (3)	N1—C41—C42	118.5 (7)
C10—C11—H11	120.0	N1—C41—C40	121.4 (8)
C12—C11—H11	120.0	C42—C41—C40	120.1 (7)
C11—C12—C7	120 (4)	N2—C42—C43	122.1 (8)
C11—C12—H12	120.0	N2—C42—C41	118.3 (7)
C7—C12—H12	120.0	C43—C42—C41	119.5 (8)
C3'—C2'—C1	110 (3)	C44—C43—C42	117.3 (8)
C3'—C2'—H2'1	109.7	C44—C43—C48	123.7 (9)
C1—C2'—H2'1	109.7	C42—C43—C48	119.0 (9)
C3'—C2'—H2'2	109.7	C45—C44—C43	120.7 (9)
C1—C2'—H2'2	109.7	C45—C44—H44	119.6
H2'1—C2'—H2'2	108.2	C43—C44—H44	119.6
C4'—C3'—C8'	120 (7)	C44—C45—C46	118.1 (9)
C4'—C3'—C2'	118 (5)	C44—C45—H45	120.9
C8'—C3'—C2'	122 (6)	C46—C45—H45	120.9
C3'—C4'—C5'	120 (8)	N2—C46—C45	124.0 (9)
C3'—C4'—H4'	120.0	N2—C46—H46	118.0
C5'—C4'—H4'	120.0	C45—C46—H46	118.0
C6'—C5'—C4'	120 (8)	C48—C47—C40	121.7 (9)
C6'—C5'—H5'	120.0	C48—C47—H47	119.1
C4'—C5'—H5'	120.0	C40—C47—H47	119.1
C7'—C6'—C5'	120 (8)	C47—C48—C43	121.5 (9)
C7'—C6'—H6'	120.0	C47—C48—H48	119.3
C5'—C6'—H6'	120.0	C43—C48—H48	119.3
C6'—C7'—C12'	120 (6)		
			
O3^i^—Sm1—N1—C37	−26.9 (7)	C12—C7—C8—C9	0(5)
O1—Sm1—N1—C37	170.6 (5)	C6—C7—C8—C9	180 (3)
O2^i^—Sm1—N1—C37	17.6 (6)	C3—C8—C9—C10	180 (2)
O6—Sm1—N1—C37	−65.3 (6)	C7—C8—C9—C10	0(4)
O5—Sm1—N1—C37	−111.4 (6)	C8—C9—C10—C11	0(4)
O4—Sm1—N1—C37	107.9 (6)	C9—C10—C11—C12	0(3)
O3—Sm1—N1—C37	78.1 (6)	C10—C11—C12—C7	0(4)
N2—Sm1—N1—C37	−177.2 (6)	C8—C7—C12—C11	0(6)
Eu1^i^—Sm1—N1—C37	43.1 (7)	C6—C7—C12—C11	180 (3)
Sm1^i^—Sm1—N1—C37	43.1 (7)	O2—C1—C2'—C3'	39 (4)
O3^i^—Sm1—N1—C41	148.1 (5)	O1—C1—C2'—C3'	−165 (3)
O1—Sm1—N1—C41	−14.4 (7)	C2—C1—C2'—C3'	−70 (3)
O2^i^—Sm1—N1—C41	−167.3 (6)	C1—C2'—C3'—C4'	−120 (4)
O6—Sm1—N1—C41	109.8 (6)	C1—C2'—C3'—C8'	66 (5)
O5—Sm1—N1—C41	63.6 (5)	C8'—C3'—C4'—C5'	0(8)
O4—Sm1—N1—C41	−77.1 (5)	C2'—C3'—C4'—C5'	−175 (4)
O3—Sm1—N1—C41	−106.8 (5)	C3'—C4'—C5'—C6'	0(9)
N2—Sm1—N1—C41	−2.2 (5)	C4'—C5'—C6'—C7'	0(11)
Eu1^i^—Sm1—N1—C41	−141.9 (4)	C5'—C6'—C7'—C12'	180 (6)
Sm1^i^—Sm1—N1—C41	−141.9 (4)	C5'—C6'—C7'—C8'	0(10)
O3^i^—Sm1—N2—C46	28.9 (8)	C4'—C3'—C8'—C9'	180 (5)
O1—Sm1—N2—C46	−9.2 (6)	C2'—C3'—C8'—C9'	−5(8)
O2^i^—Sm1—N2—C46	−158.9 (6)	C4'—C3'—C8'—C7'	0(8)
O6—Sm1—N2—C46	116.3 (6)	C2'—C3'—C8'—C7'	175 (4)
O5—Sm1—N2—C46	70.9 (6)	C6'—C7'—C8'—C3'	0(9)
O4—Sm1—N2—C46	−95.3 (6)	C12'—C7'—C8'—C3'	−180 (5)
O3—Sm1—N2—C46	−68.6 (6)	C6'—C7'—C8'—C9'	−180 (6)
N1—Sm1—N2—C46	179.0 (7)	C12'—C7'—C8'—C9'	0(8)
Eu1^i^—Sm1—N2—C46	−36.7 (7)	C3'—C8'—C9'—C10'	180 (4)
Sm1^i^—Sm1—N2—C46	−36.7 (7)	C7'—C8'—C9'—C10'	0(8)
O3^i^—Sm1—N2—C42	−148.3 (5)	C8'—C9'—C10'—C11'	0(6)
O1—Sm1—N2—C42	173.6 (6)	C9'—C10'—C11'—C12'	0(5)
O2^i^—Sm1—N2—C42	23.9 (7)	C6'—C7'—C12'—C11'	180 (5)
O6—Sm1—N2—C42	−60.8 (6)	C8'—C7'—C12'—C11'	0(7)
O5—Sm1—N2—C42	−106.2 (6)	C10'—C11'—C12'—C7'	0(6)
O4—Sm1—N2—C42	87.6 (5)	Sm1—O4—C13—O3	14.5 (8)
O3—Sm1—N2—C42	114.2 (5)	Sm1—O4—C13—C14	−162.5 (7)
N1—Sm1—N2—C42	1.9 (5)	Eu1^i^—O3—C13—O4	−162.9 (10)
Eu1^i^—Sm1—N2—C42	146.1 (4)	Sm1^i^—O3—C13—O4	−162.9 (10)
Sm1^i^—Sm1—N2—C42	146.1 (4)	Sm1—O3—C13—O4	−14.1 (7)
O3^i^—Sm1—O1—C1	27.9 (9)	Eu1^i^—O3—C13—C14	14.2 (18)
O2^i^—Sm1—O1—C1	−23.1 (10)	Sm1^i^—O3—C13—C14	14.2 (18)
O6—Sm1—O1—C1	94.4 (9)	Sm1—O3—C13—C14	163.0 (6)
O5—Sm1—O1—C1	112.0 (9)	O4—C13—C14—C15	−6.8 (13)
O4—Sm1—O1—C1	−101.2 (9)	O3—C13—C14—C15	176.1 (8)
O3—Sm1—O1—C1	−49.8 (9)	C13—C14—C15—C16	−112.2 (10)
N1—Sm1—O1—C1	−162.4 (8)	C13—C14—C15—C20	73.3 (12)
N2—Sm1—O1—C1	−173.5 (9)	C20—C15—C16—C17	−2.3 (17)
Eu1^i^—Sm1—O1—C1	−12.2 (9)	C14—C15—C16—C17	−177.0 (11)
Sm1^i^—Sm1—O1—C1	−12.2 (9)	C15—C16—C17—C18	0.5 (19)
O3^i^—Sm1—O3—C13	−169.2 (5)	C16—C17—C18—C19	1(2)
O1—Sm1—O3—C13	−89.1 (4)	C17—C18—C19—C24	178.0 (12)
O2^i^—Sm1—O3—C13	109.8 (4)	C17—C18—C19—C20	−0.5 (18)
O6—Sm1—O3—C13	146.0 (4)	C16—C15—C20—C21	−177.5 (10)
O5—Sm1—O3—C13	−118.4 (5)	C14—C15—C20—C21	−2.9 (14)
O4—Sm1—O3—C13	7.6 (4)	C16—C15—C20—C19	2.7 (14)
N1—Sm1—O3—C13	46.1 (5)	C14—C15—C20—C19	177.2 (8)
N2—Sm1—O3—C13	−25.4 (5)	C18—C19—C20—C21	178.9 (10)
Eu1^i^—Sm1—O3—C13	−169.2 (5)	C24—C19—C20—C21	0.3 (15)
Sm1^i^—Sm1—O3—C13	−169.2 (5)	C18—C19—C20—C15	−1.3 (15)
O3^i^—Sm1—O3—Eu1^i^	0.0	C24—C19—C20—C15	−179.9 (9)
O1—Sm1—O3—Eu1^i^	80.2 (2)	C15—C20—C21—C22	−180.0 (10)
O2^i^—Sm1—O3—Eu1^i^	−81.0 (2)	C19—C20—C21—C22	−0.1 (14)
O6—Sm1—O3—Eu1^i^	−44.7 (4)	C20—C21—C22—C23	0.7 (16)
O5—Sm1—O3—Eu1^i^	50.8 (3)	C21—C22—C23—C24	−1.6 (19)
O4—Sm1—O3—Eu1^i^	176.8 (3)	C22—C23—C24—C19	2(2)
N1—Sm1—O3—Eu1^i^	−144.7 (2)	C18—C19—C24—C23	−179.6 (12)
N2—Sm1—O3—Eu1^i^	143.8 (2)	C20—C19—C24—C23	−1.1 (17)
Sm1^i^—Sm1—O3—Eu1^i^	0.0	Sm1—O5—C25—O6	4.5 (8)
O3^i^—Sm1—O3—Sm1^i^	0.0	Sm1—O5—C25—C26	−176.2 (7)
O1—Sm1—O3—Sm1^i^	80.2 (2)	Sm1—O6—C25—O5	−4.6 (8)
O2^i^—Sm1—O3—Sm1^i^	−81.0 (2)	Sm1—O6—C25—C26	176.1 (6)
O6—Sm1—O3—Sm1^i^	−44.7 (4)	O5—C25—C26—C27	89.4 (10)
O5—Sm1—O3—Sm1^i^	50.8 (3)	O6—C25—C26—C27	−91.3 (10)
O4—Sm1—O3—Sm1^i^	176.8 (3)	C25—C26—C27—C28	−7.3 (13)
N1—Sm1—O3—Sm1^i^	−144.7 (2)	C25—C26—C27—C32	172.3 (8)
N2—Sm1—O3—Sm1^i^	143.8 (2)	C32—C27—C28—C29	−0.9 (15)
Eu1^i^—Sm1—O3—Sm1^i^	0.0	C26—C27—C28—C29	178.7 (9)
O3^i^—Sm1—O4—C13	−4.1 (5)	C27—C28—C29—C30	1.3 (16)
O1—Sm1—O4—C13	61.9 (5)	C28—C29—C30—C31	−1.0 (16)
O2^i^—Sm1—O4—C13	−76.1 (5)	C29—C30—C31—C32	0.2 (15)
O6—Sm1—O4—C13	−142.1 (5)	C29—C30—C31—C36	−178.1 (10)
O5—Sm1—O4—C13	119.6 (5)	C30—C31—C32—C33	−179.0 (9)
O3—Sm1—O4—C13	−7.8 (4)	C36—C31—C32—C33	−0.6 (13)
N1—Sm1—O4—C13	−154.4 (5)	C30—C31—C32—C27	0.2 (13)
N2—Sm1—O4—C13	140.8 (5)	C36—C31—C32—C27	178.6 (9)
Eu1^i^—Sm1—O4—C13	−6.0 (4)	C28—C27—C32—C33	179.4 (9)
Sm1^i^—Sm1—O4—C13	−6.0 (4)	C26—C27—C32—C33	−0.3 (13)
O3^i^—Sm1—O5—C25	−87.8 (5)	C28—C27—C32—C31	0.2 (13)
O1—Sm1—O5—C25	−164.7 (5)	C26—C27—C32—C31	−179.4 (8)
O2^i^—Sm1—O5—C25	−23.4 (6)	C31—C32—C33—C34	−0.3 (14)
O6—Sm1—O5—C25	−2.6 (4)	C27—C32—C33—C34	−179.5 (9)
O4—Sm1—O5—C25	136.0 (4)	C32—C33—C34—C35	0.9 (16)
O3—Sm1—O5—C25	−136.6 (5)	C33—C34—C35—C36	−0.4 (18)
N1—Sm1—O5—C25	56.9 (5)	C34—C35—C36—C31	−0.6 (18)
N2—Sm1—O5—C25	115.0 (5)	C30—C31—C36—C35	179.4 (10)
Eu1^i^—Sm1—O5—C25	−107.3 (5)	C32—C31—C36—C35	1.1 (15)
Sm1^i^—Sm1—O5—C25	−107.3 (5)	C41—N1—C37—C38	1.6 (12)
O3^i^—Sm1—O6—C25	88.3 (5)	Sm1—N1—C37—C38	176.7 (6)
O1—Sm1—O6—C25	24.3 (5)	N1—C37—C38—C39	−0.3 (13)
O2^i^—Sm1—O6—C25	166.3 (5)	C37—C38—C39—C40	−1.1 (14)
O5—Sm1—O6—C25	2.5 (4)	C38—C39—C40—C47	−178.5 (8)
O4—Sm1—O6—C25	−125.6 (5)	C38—C39—C40—C41	1.0 (13)
O3—Sm1—O6—C25	131.5 (5)	C37—N1—C41—C42	177.7 (7)
N1—Sm1—O6—C25	−112.8 (5)	Sm1—N1—C41—C42	2.5 (9)
N2—Sm1—O6—C25	−56.3 (5)	C37—N1—C41—C40	−1.6 (11)
Eu1^i^—Sm1—O6—C25	104.8 (4)	Sm1—N1—C41—C40	−176.8 (5)
Sm1^i^—Sm1—O6—C25	104.8 (4)	C39—C40—C41—N1	0.4 (11)
Sm1^i^—O2—C1—O1	11.3 (16)	C47—C40—C41—N1	179.9 (7)
Eu1^i^—O2—C1—O1	11.3 (16)	C39—C40—C41—C42	−178.9 (7)
Sm1^i^—O2—C1—C2	−151.7 (11)	C47—C40—C41—C42	0.6 (11)
Eu1^i^—O2—C1—C2	−151.7 (11)	C46—N2—C42—C43	0.0 (11)
Sm1^i^—O2—C1—C2'	163.1 (15)	Sm1—N2—C42—C43	177.3 (6)
Eu1^i^—O2—C1—C2'	163.1 (15)	C46—N2—C42—C41	−178.8 (7)
Sm1—O1—C1—O2	7.5 (17)	Sm1—N2—C42—C41	−1.5 (9)
Sm1—O1—C1—C2	171.7 (10)	N1—C41—C42—N2	−0.6 (10)
Sm1—O1—C1—C2'	−144.1 (16)	C40—C41—C42—N2	178.7 (7)
O2—C1—C2—C3	−6(3)	N1—C41—C42—C43	−179.5 (7)
O1—C1—C2—C3	−172 (2)	C40—C41—C42—C43	−0.1 (11)
C2'—C1—C2—C3	86 (3)	N2—C42—C43—C44	0.1 (12)
C1—C2—C3—C4	95 (3)	C41—C42—C43—C44	178.9 (7)
C1—C2—C3—C8	−82 (3)	N2—C42—C43—C48	−179.9 (7)
C8—C3—C4—C5	0(4)	C41—C42—C43—C48	−1.2 (12)
C2—C3—C4—C5	−177 (2)	C42—C43—C44—C45	−0.3 (13)
C3—C4—C5—C6	0(4)	C48—C43—C44—C45	179.8 (9)
C4—C5—C6—C7	0(5)	C43—C44—C45—C46	0.3 (14)
C5—C6—C7—C8	0(6)	C42—N2—C46—C45	−0.1 (12)
C5—C6—C7—C12	180 (3)	Sm1—N2—C46—C45	−177.3 (7)
C4—C3—C8—C7	0(4)	C44—C45—C46—N2	−0.1 (14)
C2—C3—C8—C7	177 (3)	C39—C40—C47—C48	179.8 (9)
C4—C3—C8—C9	180 (2)	C41—C40—C47—C48	0.3 (13)
C2—C3—C8—C9	−3(3)	C40—C47—C48—C43	−1.6 (14)
C12—C7—C8—C3	−180 (3)	C44—C43—C48—C47	−178.0 (9)
C6—C7—C8—C3	0(6)	C42—C43—C48—C47	2.1 (13)

Symmetry codes: (i) −*x*+2, −*y*+2, −*z*+2.

### Hydrogen-bond geometry (Å, °)

**Table:**

*D*—H···*A*	*D*—H	H···*A*	*D*···*A*	*D*—H···*A*
C37—H37···O2^i^	0.93	2.45	3.097 (9)	127
C46—H46···O1	0.93	2.38	3.047 (10)	129
C11—H11···O5^ii^	0.93	2.48	3.38 (2)	163
C47—H47···O4^iii^	0.93	2.50	3.352 (10)	153
C35—H35···O6^iv^	0.93	2.67	3.327 (12)	128
C16—H16···Cg1^v^	0.93	2.87	3.66 (3)	144
C39—H39···Cg2^iii^	0.93	2.83	3.673 (18)	151

Symmetry codes: (i) −*x*+2, −*y*+2, −*z*+2; (ii) −*x*+1, −*y*+2, −*z*+2; (iii) −*x*+3, −*y*+1, −*z*+2; (iv) −*x*+2, −*y*+1, −*z*+3; (v) *x*+1, *y*, *z*.
